# The Time Course of Compensatory Puffing With an Electronic Cigarette: Secondary Analysis of Real-World Puffing Data With High and Low Nicotine Concentration Under Fixed and Adjustable Power Settings

**DOI:** 10.1093/ntr/ntab013

**Published:** 2021-01-23

**Authors:** Sharon Cox, Maciej L Goniewicz, Leon Kosmider, Hayden McRobbie, Catherine Kimber, Lynne Dawkins

**Affiliations:** 1 Department of Behavioural Science and Health, University College London, London, UK; 2 Department of Health Behavior, Roswell Park Comprehensive Cancer Center, Buffalo, NY, USA; 3 Department of General and Inorganic Chemistry, Medical University of Silesia, Katowice FOPS in Sosnowiec, Sosnowiec, Poland; 4 National Drug and Alcohol Research Centre, University of New South Wales, Sydney, Australia; 5 Centre for Addictive Behaviours Research, London South Bank University, London, UK

## Abstract

**Introduction:**

In a secondary analysis of our published data demonstrating compensatory vaping behavior (increased puff number, puff duration, and device power) with e-cigarettes refilled with low versus high nicotine concentration e-liquid, here we examine 5-day time course over which compensatory behavior occurs under fixed and adjustable power settings.

**Aims and Methods:**

Nineteen experienced vapers (37.90 ± 10.66 years, eight females) vaped ad libitum for 5 consecutive days under four counterbalanced conditions (ie, 20 days in total): (1) low nicotine (6 mg/mL)/fixed power (4.0 V/10 W); (2) low nicotine/adjustable power; (3) high nicotine (18 mg/mL)/fixed power; (4) high nicotine/adjustable power (at 1.6 Ohm). Puff number, puff duration, and power settings were recorded by the device. For each day, total daily puffing time was calculated by multiplying daily puff number by mean daily puff duration.

**Results:**

A significant day × setting interaction revealed that whilst puffing compensation (daily puffing time) continued to increase over 5 days under fixed power, it remained stable when power settings were adjustable. Separate analysis for puff number and puff duration suggested that the puffing compensatory behavior was largely maintained via longer puff duration.

**Conclusions:**

Under fixed power conditions (4.0 V/10 W), vapers appear to compensate for poor nicotine delivery by taking longer puffs and this compensatory puffing appears to be maintained over time.

**Implications:**

Studies in smokers suggest that when switching to lower nicotine levels, compensation for poorer nicotine delivery is transient. Our novel findings suggest that vapers show a different pattern of compensation which is influenced by both nicotine strength and device power settings. When power is fixed (4.0 V; 10 W), compensation (via more intensive puffing) appears prolonged, persisting up to 5 days. Under adjustable settings when power is increased, puffing patterns remain stable over time. Implications of such compensatory behaviors for product safety and user satisfaction need further exploration.

## Introduction

Nicotine is widely considered to be the primary motivation for smoking or vaping (using an e-cigarette). Continual and intermittent use of nicotine enables users to alleviate withdrawal symptoms which in turn reinforces behavior and maintains use. The amount of nicotine contained in cigarettes or e-liquid has a large impact on how users either smoke or vape (eg, puff time, duration, and volume). Compensatory behavior is well documented in smokers,^1,2^ and more recently evidenced in vapers (e-cigarette users).^3–5^

In conditions where smokers use very low nicotine or non-nicotine cigarettes, they adjust their puffing behavior to compensate for this reduction to maintain stable blood nicotine levels.^1,6,7^ Smokers who had been given cigarettes with reduced nicotine content took longer and deeper puffs, and increased the number of puffs taken and decreased the time in between puffs.^1^ This compensatory behavior is linked to a reduction in satisfaction and can also be associated with greater toxicant exposure. For example, Strasser et al.’s^8^ laboratory-based study showed that staged nicotine reductions in cigarettes—from 0.6 to 0.3, and eventually to 0.05—led to an increase in total puff volume which in turn resulted in higher levels of exhaled carbon monoxide in the 0.3 and 0.05 conditions compared with the 0.6 condition. However, larger and more recent trial evidence by Donny et al.^2^ suggests that negative effects (ie, compensatory puffing) can be lessened if nicotine is adequately reduced over a longer timeframe than is typically used in laboratory studies.

To counter the concerns that smoking lower nicotine containing cigarettes can lead to greater harm, there is some evidence that compensatory behavior is transient, and that attempts to self-titrate is not maintained. Evidence by MacQueen et al.^9^ showed that after 12 hours of abstinence, smokers using both, nicotine containing cigarettes and placebo cigarettes (Quest brand) ad libitum, took larger, longer, and more frequent puffs with the latter compared with the former. However, this was only observed in the first and second bouts of exposure and total puff volume had decreased after the third and was almost abolished by the fourth round of exposure. Therefore, compensatory puffing pattern is positively skewed to early phases on transitioning only, and could eventually result in a reduction in smoking and ultimately cessation.

To date, the majority of the evidence for compensatory puffing derives from cigarette smoking. Patterns of compensatory vaping behavior are only just beginning to emerge^3–5,10,11^ and whether compensatory vaping behavior is transient as MacQueen et al.^9^ demonstrated with cigarette smoking is unknown. Evidence by Farsalinos et al.^11^ shows that vapers can compensate by way of increasing the device power settings in lieu of adjusting their puffing patterns; this suggests that vapers can use lower nicotine concentration e-liquids and still receive optimal nicotine hits. In our recent work,^3^ we showed that amongst 19 experienced vapers, the use of a lower nicotine concentration (6 mg/mL) e-liquid for 1 week under fixed-device settings was associated with a greater number of puffs, longer puff duration and shorter interpuff interval compared with the use of a higher nicotine concentration (18 mg/mL) e-liquid under fixed or adjustable power settings. Our participants also demonstrated a modest preference for higher power settings under lower nicotine concentration e-liquid conditions when they were able to adjust the device settings (low nicotine, 4.5 V [12.66 W] vs. high nicotine, 4.3 V [11.56 W]). Conversely, although yet to be demonstrated empirically, it is likely that if an experienced user switches to a device containing a higher nicotine concentration e-liquid, such as the 59 mg/mL nicotine JUUL or other pod-based device available in the United States, puff number and puff duration would be expected to decrease.

In our previous study,^3^ we did not explore in detail the differences in daily pattern of compensation, instead we report the weekly average of user behavior across each condition. Thus, we did not examine if, like the MacQueen et al. study,^9^ participants’ compensation was positively skewed to the early stages of adjustment to lower nicotine concentration and this is important as data on the transitory nature of compensation are scarce. The aim of the current paper is to explore the dynamics of compensatory puffing behavior (ie, number of puffs and puff duration) as observed in the vapers from our earlier study across 5 consecutive days in each of the four conditions. Furthermore, we explore in finer detail how vapers manipulate their device settings to compensate in the lower nicotine concentration conditions.

Here, we present a secondary data analysis taken from our Dawkins et al. study,^3^ using experienced vapers’ “real-world” (outside of the laboratory) puffing data using a third-generation device (eVic Supreme) over 4 weeks with participants being able to use the device “in their usual context” across two nicotine conditions (higher vs. lower nicotine concentration) and two device settings (fixed-device vs. adjustable). The data presented here are a closer examination of the puffing data over 5 consecutive days in each condition. Having previously demonstrated compensatory behavior with lower versus higher nicotine concentration e-liquid (increased puff number, puff duration, and power) in our previous study,^3^ here we were particularly interested in exploring the time course over which this compensatory behavior occurred and whether it diminishes.

## Methods

A more detailed description of our methodology, including sample size calculation and participant demographics can be found in our previously published work (full study protocol,^12^ behavioral and biomarker analyses^3^). The original data file is publicly available (https://openresearch.lsbu.ac.uk/item/86z7w).

### Design and Ethical Approval

Ethical approval was granted by London South Bank University ethics committee (UREC 1604) and conducted in accordance with the ethical standards of the 1964 Declaration of Helsinki.

A repeated measures counterbalanced design with four conditions was used: low (6 mg/mL) nicotine/fixed power; high (18 mg/mL) nicotine/fixed power; low nicotine/adjustable power; high nicotine/adjustable power. Puffing was measured over 5 consecutive days in each condition (ie, the first and last days of each week were excluded as these represented crossover days between conditions). All participants started on fixed conditions therefore only nicotine e-liquid strengths (low and high) were counterbalanced. Within the analysis this counterbalancing of conditions is referred to as order. Supplementary Figure 1 presents the study design and flow.

### Participants

Nineteen daily and exclusive vapers (verified by exhaled CO), *M* age = 37.90 years (*SD* = 10.66), eight females, 18 participants were white British and one biracial British, all participants had completed at least senior school (high school) education, one participant was retired and all others were in employment. Months since smoking quit date was 25.95 (*SD* = 25.35). Mean baseline salivary cotinine was 324.08 (*SD* = 219.45). Twelve participants used a rechargeable (non-cigalike) second-generation e-cigarette and eight used a rechargeable modular tank device. Some participants used more than one type of e-cigarette per day.

### Measures and Procedure

Participants met with the researcher on five separate occasions (at baseline and the end of each of the four experimental conditions). At baseline participants provided written informed consent, demographic characteristics, and smoking/vaping history then sampled four e-liquids (tobacco, fruit, bakery, and menthol flavors) selecting one to be used for the next 4 weeks. Participants sampled the flavors with the e-liquid strength they were going to be using in the following week. Nicotine e-liquid strength was counterbalanced over the 4-week period (Supplementary Figure 1). Participants were provided with an eVic Supreme by Joyetech fitted with a “Nautilus Aspire” tank housing a BVC atomizer (1.6 Ohm) and seven 10 mL bottles of e-liquid for the week (6 or 18 mg/mL according to condition). Subjective effects, urine and saliva samples were collected at the end of every week as part of the outcomes for the main study^3^; these are not reported here.

To ensure device familiarity before changes were permitted, for the first 2 weeks participants were always asked to start on the fixed (4.0 V/10 W) device setting. Changes to voltage were permitted during the last 2 weeks. Voltage could be adjusted (between 3.0 and 6.0 V) by turning a dial under the display unit on the eVic. Given that the atomizer resistance was fixed at 1.6 Ohm, adjusting the voltage upwards resulted in increased wattage (overall power output). Participants could adjust the airflow but nobody reported doing this, participants were asked to not use their own devices and e-liquids for the duration of the study. At the end of each condition, puffing and power data were downloaded into Excel from the device using myVapor software, upon downloading the data file this was scanned by two of researchers (SC and LK) for any irregularities and there were no instances of participants adjusting the power in the fixed condition.

Daily puffing topography was measured by the device, including total daily puff number and puff duration (in seconds). Reported here are the e-cigarette usage data recorded by the eVic, this includes time of the puff (not used in this analysis), puff length (in seconds), atomizer resistance, voltage, and wattage.

### Data Preparation and Analysis

Each condition was designed to last for 7 days, but here and in our previously published paper,^3^ we exclude the first day from the analysis because this reflected the participants’ familiarity and adjustment to the device and setting (reducing potential carry-over effects between device and nicotine conditions). We also excluded data collected during last day since during that day the condition had changed. After excluding first and last days, we analyzed complete sets of data collected over 5 days. All button presses <1 second (false button presses or non-starts) were deleted.

*Total puffing time* for each participant, each day, under fixed and adjustable, and high and low nicotine concentrations was computed by multiplying the number of puffs per day by daily average puff duration. For each participant, a *puffing compensation score* was then calculated under fixed and adjustable settings for each day by subtracting the daily total puffing time for the high condition from the total puffing time for corresponding day in the low condition. The higher the positive value is indicative of greater compensation (ie, more puffing) with low versus high nicotine concentration.

Prior to analysis, frequency distributions and stem and leaf plots for normality and outliers respectively were computed. Fixed settings all showed a slight positive skew. Adjustable setting variables were either normal or showed slight positive skew. Stem and leaf plots indicated some extreme outliers (six outliers: in the fixed condition, one at day 1 and one at day 2; in the adjustable condition, one at day 1, two at day 4, and one at day 5. No participants were outliers across multiple variables) at the upper end. Transformations failed to normalize the data (sqr root, log, and cube root). Therefore, to reduce the influence of the six outliers these values were reassigned a score that was 1 unit higher than the next highest score (as per Tabachnick and Fidell, p. 69). Following reviewer feedback, we also present data before these outliers are removed (Supplementary Figure 2).

Using data with outliers reduced, a repeated measure ANOVA, with two settings (Fixed and Adjustable) × 5 days (1–5) as within subject factors and × 4 order (nicotine strength order) as a between subject factor. Mauchly’s test of sphericity was violated for day so we used the Greenhouse–Geisser output. For the adjustable condition only, a change score was computed by subtracting the wattage for each day (1–5) for high 18 mg/mL nicotine from low 6 mg/mL. Lastly, in order to explore whether compensation continued across 5 days of vaping, simple contrasts were used to compare puffing compensation score at days 2, 3, 4, and 5 with day 1 (ie, the first day of vaping under that particular condition). All analyses were conducted using SPSS (v26).

## Results

**Figure 1.**
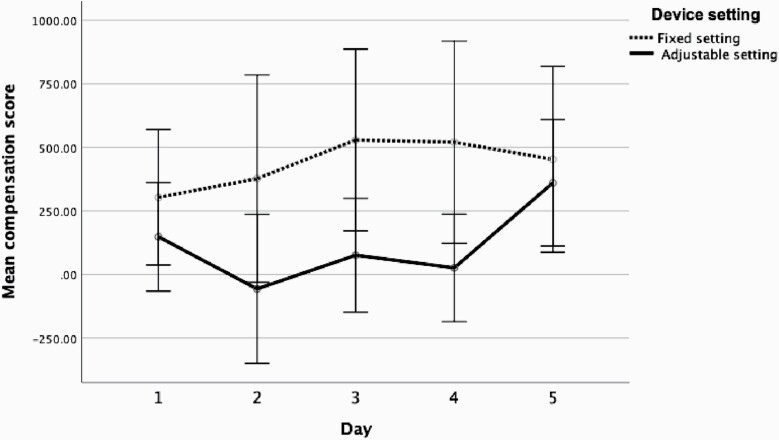
Daily puffing compensation score (puff number × mean puff duration; PCS) across days (1–5) under fixed and adjustable power settings. Bars represent the 95% confidence interval.

Analysis of the puffing compensation score (difference in total puffing time in the high nicotine condition subtracted from the low nicotine condition for each day) showed the main effect of day was not statistically significant (*F*(1.94, 40) = 1.83, *p* = .18). However, there was a significant main effect of setting (*F*(1, 10) = 5.71, *p* = .04), and a statistically significant day × setting interaction (*F*(3.19, 31.48) = 2.47, *p* = .04). There were no significant interactions with order. As shown in Figure 1, users’ puffing compensation (more intensive puffing in the low vs. high nicotine condition) increased across days under fixed but not under adjustable power settings.

Separate repeated measures ANOVAs for fixed and adjustable with day as the within subject factor and order as between subject factor with simple contrasts were used to compare puffing compensation score at days 2, 3, 4, and 5 with day 1. For adjustable, none of the comparisons were statistically significant (all *p*s > .05). There was no main effect of order within either analysis *p* > .05. For fixed setting, with the exception of days 1 and 2, each comparison was statistically significant (*p* < .05) (Supplementary Table 1).

**Figure 2.**
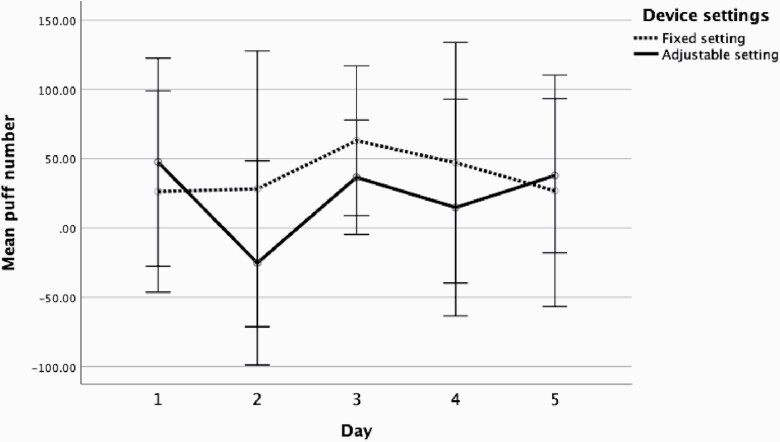
Mean puff number (calculated by difference score low − high) across 5 days with fixed and adjustable setting. Bars represent the 95% confidence interval.

**Figure 3.**
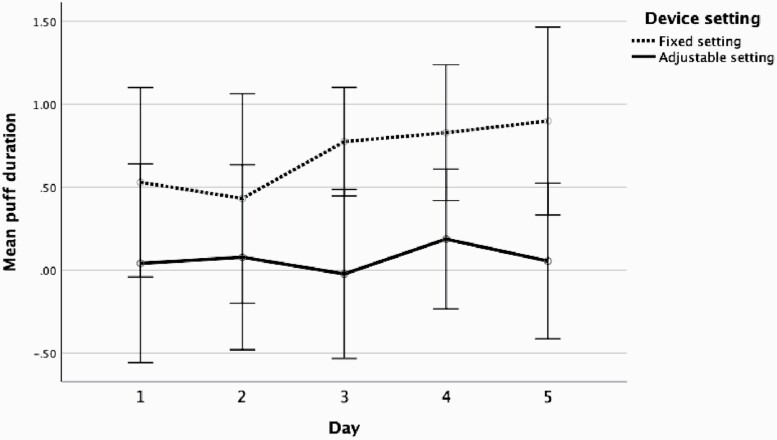
Mean puff duration (calculated by difference score low − high) across 5 days with fixed and adjustable power. Bars represent the 95% confidence interval.

To ascertain the most significant factor driving puffing compensation, puff number and duration were separated, and difference scores were computed by subtracting daily puff number and average daily puff duration from the high nicotine condition from the corresponding day in the low nicotine condition. These scores are plotted for both fixed and adjustable conditions across the 5 days in Figure 2 (puff number) and Figure 3 (puff duration). Figures 2 and 3 both show that users puffed more frequently and for an increased duration with fixed settings than adjustable. Exploring this further, separate repeated measures ANOVAs for fixed and adjustable with day as the within subject factor and order as a between subject factor with simple contrasts were used to compare puff number (Supplementary Table 2) and puff duration (Supplementary Table 3) means at days 2, 3, 4, and 5 with day 1. Puff duration significantly differed between days 1 and 2 under adjustable conditions. Again, across the analyses there was no main effect of order *p* > .05.

**Figure 4.**
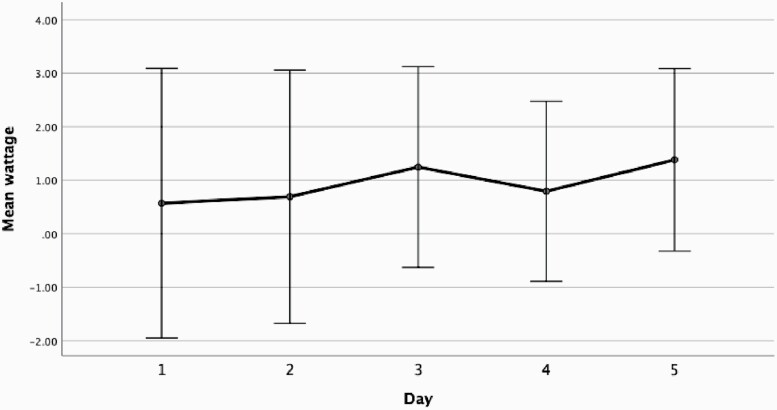
Mean wattage changes over days 1–5 in the adjustable power condition (wattage change score calculated by subtracting the wattage for each day [1–5]) for 18 mg/mL nicotine from 6 mg/mL. In the fixed condition, there was no wattage change because the power was always fixed 4 V/10 W. Bars represent the 95% confidence interval.

For the adjustable condition, we also computed power change scores by subtracting the wattage for each day (1–5) for high from low. A repeated measures ANOVA 5 × day and × 4 order was run on these change scores with simple contrasts to compare days 2, 3, 4, and 5 with day 1. Visual inspection of the mean daily changes in wattage between the high and low nicotine conditions in Figure 4 shows a trend toward a linear increase in power over the 5 days but there was no statistically significant main effect (*F*(1.40, 12.56) = 0.60, *p* = .50) or significant contrasts (all *p*s > .05).

## Discussion

Here we analyzed the time course of compensatory behavior (more intensive puffing with low vs. high nicotine strength) under fixed and adjustable power settings in order to explore whether compensation is transient or persists over 5 consecutive days.

Overall, we show that there is a clear distinction in compensatory behavior with greater total puffing time in the fixed compared with the adjustable setting condition. Over the 5-day time period, participants demonstrated greater effort to compensate via increased puff number and puff duration under fixed compared with adjustable power settings. As our earlier work showed,^3^ greater compensation results in higher levels of liquid consumed and exposure to more toxicants emitted from the aerosol. Switching to a lower nicotine e-liquid without altering power settings may be off-putting for vapers, who may find it more effortful to vape more to achieve stable blood nicotine levels and satisfaction.

Furthermore, under fixed and adjustable power settings we observed a changing pattern in compensation over 5 days. Under fixed conditions vapers continued to compensate by increasing total puff time compared with baseline over 5 consecutive days, whereas under adjustable settings, the pattern of compensation was more variable with no clear evidence of compensation through puffing behavior. Consistent with previous reports,^11^ the data suggest that when power is adjustable, compensation appears to be primarily driven through adjusting the power upwards, although we do not have statistically significant evidence to support this. This may be due to the sample size and also the variation between subjects. Nonetheless, adjustable power settings may help e-cigarette vapers to satisfy craving and yield greater satisfaction, especially in the absence of higher strength nicotine.

This analysis shows that compensatory behavior differs according to device settings but also differs to cigarette smoking. Compensatory behavior associated with smoking reduced nicotine containing cigarettes has been shown to be transient and positively skewed.^9^ Our results demonstrate that participants continued to compensate through to the fifth day of vaping (and perhaps beyond) when power could not be adjusted (ie, fixed power settings). When compensatory puffing was further examined according to puff number and puff duration, it seemed that the increase in puff duration, rather than puff number, was the primary indicator of compensation. This is consistent with earlier studies, puff duration has the most important role driving nicotine yield when comparing interpuff intervals and puffs volume.^13^ While our sample size is small, it does provide important data to suggest that theories of nicotine compensation developed within the smoking literature may be less relevant to understanding compensatory puffing behavior in e-cigarette users. Taken together with our earlier findings, it also suggests that higher, rather than lower nicotine concentration e-liquids may be preferable to reduce total puffing time, especially when power settings cannot be adjusted.

One limitation of our study is that we present only a snapshot of compensatory behavior over a 5-day period and the pattern of scores (particularly for puff duration and power) suggests that compensatory behavior might continue even beyond 5 days. A second, and related limitation is the use of “day 1” in each condition as a comparison. We selected day 1 as the “baseline” to which we compared subsequent days in order to document the persistence of compensatory behavior. However, the study was not designed specifically to analyze the time course of compensatory behavior and, as noted earlier, the four conditions ran consecutively without any washout period. The true “day 1” of the second, third, and fourth conditions therefore also constituted the last day of the previous condition (crossover days). Hence, although the “day 1” used here constituted the first full day of each condition, some compensation may have already occurred prior to this during the crossover day. This would in fact, militate against detecting a difference in compensation from day 1 to subsequent days and may explain the lack of a statistically significant difference across high and low nicotine conditions in relation to power (wattage). Any future work should therefore consider extending the number of days under each condition and use a more accurate baseline. A third limitation relates to order effects; although our conditions were counterbalanced, we always presented the fixed conditions first. It is possible therefore that the difference in compensatory scores between the fixed and adjustable power conditions may at least partly be explained by practice or carry-over effects. Encouragingly though, we found no significant main effects or interactions with order. Lastly, prior research has indicated sex differences in compensatory behavior and titration of nicotine intake,^14^ it was not possible to address this in this dataset, however this should be studied in future works where statistical power allows.

To conclude, our data suggest that compensatory puffing with lower nicotine concentration e-liquid is persistent, continuing for at least 5 consecutive days and perhaps beyond when power settings are fixed (4.0 V/10 W with 1.6 Ohm atomizer resistance). This differs from the transient pattern of compensatory puffing that has been described in cigarette smokers. Compensatory vaping behavior also appears to differ depending on e-cigarette device settings; in contrast to fixed settings, there was no evidence of compensatory puffing when power settings were adjustable.

## Supplementary Material

A Contributorship Form detailing each author’s specific involvement with this content, as well as any supplementary data, are available online at https://academic.oup.com/ntr.

ntab013_suppl_Supplementary_CRediT_taxonomy-form

ntab013_suppl_Supplementary_Figure_1

ntab013_suppl_Supplementary_Figure_2

## Funding

This study was supported by grant C50878/A21130 from Cancer Research UK. MLG was supported in part by NIH National Institute on Drug Abuse (NIDA) grant R01DA037446 and NIH National Cancer Institute (NCI)/US Food and Drug Administration (FDA) grant DA U54CA228110. Information and the views and opinions expressed in this publication are those of the author only and do not necessarily represent the views, official policy, or position of the U.S. Department of Health and Human Services or any of its affiliated institutions or agencies. SC receives salary support from Cancer Research UK (C1417/A22962).

## Declaration of Interests

SC provides expert consultancy to providers of UK life insurance. LD has conducted research for independent electronic cigarette companies. These companies had no input into the design, conduct, or write up of the projects. She has also acted as a consultant for the pharmaceutical industry and as an expert witness in a patent infringement case (2015). MLG received a research grant from Pfizer and serves on an advisory board to Johnson & Johnson, manufacturers of smoking cessation medications. HM has received honoraria for speaking at research symposia and received benefits in kind and travel support from, and has provided consultancy to, the manufacturers of smoking cessation medications. LK worked as an expert for the Polish National Committee for Standardization and for the European Committee for standardization of requirements and test methods for e-liquids and emissions. LK was also an employee of the Institute of Occupational Medicine and Environmental Health. One of the institute’s objectives is outsourcing for the industrial sector, including manufacturers of e-cigarettes. CK has no conflicts of interest to declare.
