# Supplementary material for: The Time Course of Compensatory Puffing With an Electronic Cigarette: Secondary Analysis of Real-World Puffing Data With High and Low Nicotine Concentration Under Fixed and Adjustable Power Settings
Source: Nicotine Tob Res. 2021 Jan 23;23(7):1153–9. doi: 10.1093/ntr/ntab013 (PMC8186419; doi:10.1093/ntr/ntab013)
Supplement: ntab013_suppl_Supplementary_Figure_1 [file ntab013_suppl_supplementary_figure_1.pdf]

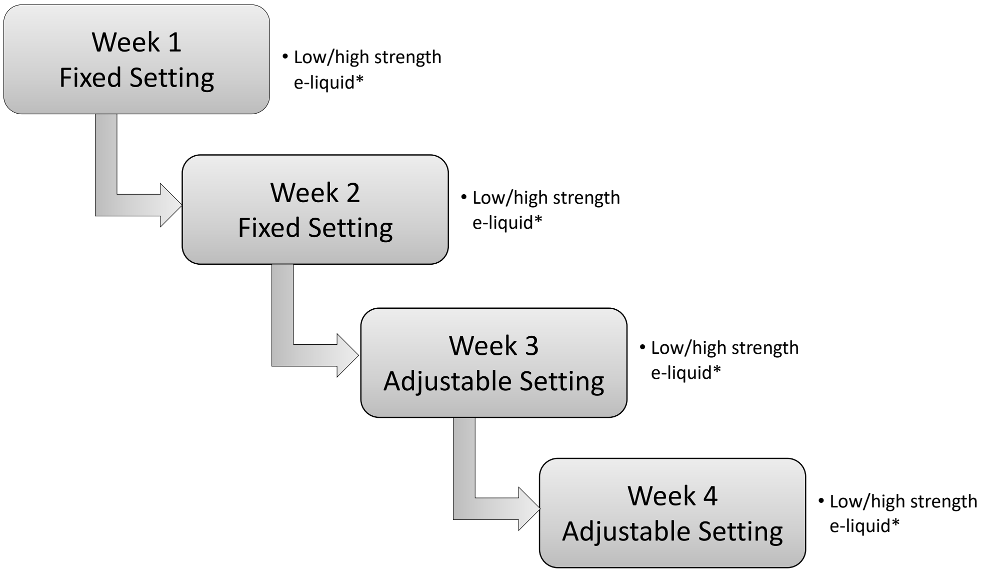

Supplementary Figure 1: Flow of participant conditions. Participants were assigned to each condition for 7 days but days 1 and 7 of each week were condition cross over days and therefore not included in the analysis. *E-liquid strength was counterbalanced.
