# Supplementary material for: The Time Course of Compensatory Puffing With an Electronic Cigarette: Secondary Analysis of Real-World Puffing Data With High and Low Nicotine Concentration Under Fixed and Adjustable Power Settings
Source: Nicotine Tob Res. 2021 Jan 23;23(7):1153–9. doi: 10.1093/ntr/ntab013 (PMC8186419; doi:10.1093/ntr/ntab013)
Supplement: ntab013_suppl_Supplementary_Figure_2 [file ntab013_suppl_supplementary_figure_2.docx]

**Supplementary Figure 2**

**
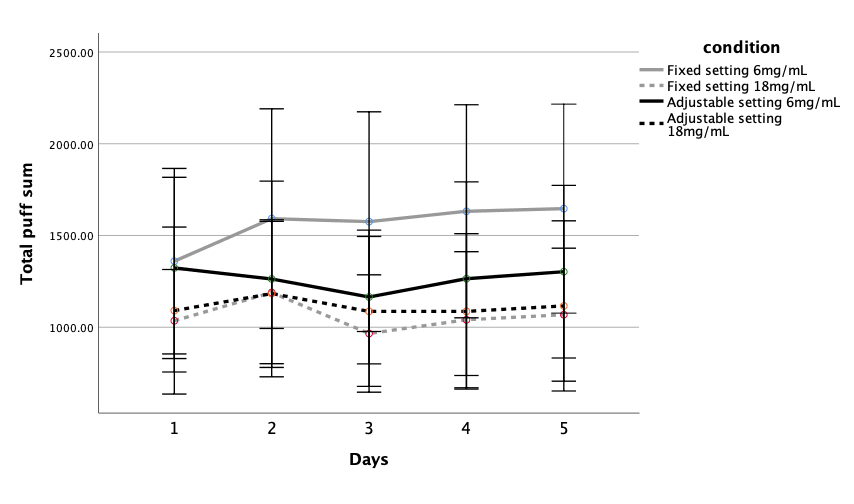
**

**Supplementary Figure 2: Daily total puff sum (puff number x puff time) across days (1-5) under fixed and adjustable power settings. Bars represent the 95% confidence interval.**

A 4 (setting x nicotine strength condition) x 5 (day) repeated measures ANOVA showed there was a significant effect of condition F(3,33) = 7.57, p = .001. There was no significant effect of day, nor an interaction of day x condition.

**Supplementary Table 1. Simple contrast of puffing compensation scores (PCS) (all compared to day 1) for fixed and adjustable settings.**

|  | **Fixed** | **Adjustable** |
| --- | --- | --- |
| **Day 1-2** | F (1,14) =3.97, *p* = 0.07 | F (1,11) = 5.48, *p* = 0.04* |
| **Day 1-3** | F (1,14) = 8.86, *p* = 0.01* | F (1,11) = 0.76, *p* = 0.40 |
| **Day 1-4** | F (1,14) = 5.99, *p* = 0.03* | F (1,11) = 2.88, *p* = 0.19 |
| **Day 1-5** | F (1,14) = 6.30, *p* = 0.03* | F (1,11) = 3.13, *p* = 0.20 |

*Indicates statistical significance p<0.05

**Supplementary Table 2. Simple contrast of puff number (all compared to day 1) for fixed and adjustable settings.**

|  | **Fixed** | **Adjustable** |
| --- | --- | --- |
| **Day 1-2** | F (1,14) = 1.69, *p* = 0.21 | F (1,9) = 13.34, *p* = 0.01* |
| **Day 1-3** | F (1,14) = 1.22, *p* = 0.29 | F (1,9) = 0.02, *p* = 0.91 |
| **Day 1-4** | F (1,14) = 0.77, *p* = 0.39 | F (1,9) = 1.35, *p* = 0.28 |
| **Day 1-5** | F (1,14) = 0.38, *p* = 0.55 | F (1,9) = 0.16, *p* = 0.70 |

*Indicates statistical significance p<0.05

**Supplementary Table 3. Simple contrast of puff duration (all compared to day 1) for fixed and adjustable settings.**

|  | **Fixed** | **Adjustable** |
| --- | --- | --- |
| **Day 1-2** | F (1,14) = 0.53, *p* = 0.48 | F (1,9) = 0.04, *p* = 0.83 |
| **Day 1-3** | F (1,14) = 3.29, *p* = 0.09 | F (1,9) = 1.17, *p* = 0.31 |
| **Day 1-4** | F (1,14) = 2.44, *p =* 0.14 | F (1,9) = 0.01, *p* = 0.98 |
| **Day 1-5** | F (1,14) = 3,01, *p* = 0.11 | F (1,9) = 0.61, *p* = 0.46 |
